# Longitudinal Bidirectional Relationships Between Maternal Depressive/Anxious Symptoms and Children's Tic Frequency in Early Adolescence

**DOI:** 10.3389/fpsyt.2021.767571

**Published:** 2021-11-24

**Authors:** Tomoko Yagi, Shuntaro Ando, Satoshi Usami, Syudo Yamasaki, Masaya Morita, Tomoki Kiyono, Noriyuki Hayashi, Kaori Endo, Yudai Iijima, Yuko Morimoto, Sho Kanata, Shinya Fujikawa, Shinsuke Koike, Yukiko Kano, Mariko Hiraiwa-Hasegawa, Atsushi Nishida, Kiyoto Kasai

**Affiliations:** ^1^Department of Child Neuropsychiatry, Graduate School of Medicine, The University of Tokyo, Tokyo, Japan; ^2^Department of Neuropsychiatry, Graduate School of Medicine, The University of Tokyo, Tokyo, Japan; ^3^Department of Psychiatry and Behavioural Sciences, Tokyo Metropolitan Institute of Medical Science, Tokyo, Japan; ^4^Graduate School of Education, The University of Tokyo, Tokyo, Japan; ^5^Department of Evolutionary Studies of Biosystems, School of Advanced Sciences, SOKENDAI (The Graduate University for Advanced Studies), Hayama, Japan; ^6^Department of Psychiatry, Teikyo University School of Medicine, Tokyo, Japan; ^7^Centre for Adolescent Health, Murdoch Children's Research Institute, Melbourne, VIC, Australia; ^8^University of Tokyo Institute for Diversity and Adaptation of Human Mind, The University of Tokyo, Tokyo, Japan; ^9^The International Research Center for Neurointelligence (WPI-IRCN), The University of Tokyo Institutes for Advanced Study (UTIAS), Tokyo, Japan

**Keywords:** tics, tic frequency, maternal depressive/anxious symptoms, longitudinal study, general population study, early adolescence

## Abstract

**Background:** Previous studies have revealed an association between maternal depressive/anxious symptoms and children's tics. However, the longitudinal relationships between these symptoms remain unclear. We examined the longitudinal relationships between maternal depressive/anxious symptoms and children's tic frequency in early adolescence with a population-based sample.

**Methods:** The participants consisted of 3,171 children and their mothers from the Tokyo Teen Cohort (TTC) study, a population-representative longitudinal study that was launched in Tokyo in 2012. Maternal depressive/anxious symptoms and children's tics were examined using self-report questionnaires at the ages of 10 (time 1, T1) and 12 (time 2, T2). A cross-lagged model was used to explore the relationships between maternal depressive/anxious symptoms and children's tic frequency.

**Results:** Higher levels of maternal depressive/anxious symptoms at T1 were related to an increased children's tic frequency at T2 (β = 0.06, *p* < 0.001). Furthermore, more frequent children's tics at T1 were positively related to maternal depressive/anxious symptoms at T2 (β = 0.06, *p* < 0.001).

**Conclusions:** These findings suggest a longitudinal bidirectional relationship between maternal depressive/anxious symptoms and children's tic frequency in early adolescence that may exacerbate each other over time and possibly create a vicious cycle. When an early adolescent has tics, it might be important to identify and treat related maternal depressive/anxious symptoms.

## Introduction

Tics are sudden, rapid, recurrent, and non-rhythmic motor movements or vocalizations. The Diagnostic and Statistical Manual of Mental Disorders, 5th edition (DSM-5) includes three tic disorders (1). Tourette syndrome (TS) is defined by the presence of at least two motor tic behaviors and one vocal tic behavior for a minimum period of a year, manifesting before the age of 18. Chronic tic disorder (CT) is defined by the presence of either motor or vocal tics for at least 1 year, while provisional tic disorder is defined as tics that have been present for less than a year. Recent population-based studies have demonstrated that tics are more common than previously recognized (2–5). According to the International Classification of Diseases 10th Revision (ICD-10), which is an international diagnostic classification developed by the World Health Organization (WHO), one in five to ten children has experienced tics (6). Tic disorders impose a psychosocial burden on children and their families because tics are characterized by the visibility of symptoms, which can cause stigma and prejudice (7–10). Attention-deficit/hyperactivity disorder (ADHD) and obsessive-compulsive disorder (OCD) are common comorbidities of tic disorders (7, 11, 12). Tic disorders tend to be remitted with age through adolescence (7, 13, 14). The overall similarity in these patterns of comorbidity and natural history among tic disorders suggests that tic disorders have etiological continuity (15–17), and a recent diagnosis of “tic spectrum disorders” has been suggested (18). Many clinical studies and experimental studies historically use tic frequency on measures to assess severity at outcome (19–24).

Tic disorders consist of a complex involvement of both multiple genes and environmental factors (25, 26). Little is known about the exact brain mechanisms associated with tic development and expression (27, 28), although preliminary evidence from neurochemical and neuroimaging investigations suggests a primary role for dysfunction of the dopaminergic pathways within the cortico-striato-cortico-frontal circuitry (29–31). Environmental factors for tics include infection and autoimmune dysfunction, maternal environment during pregnancy, and psychosocial stress (11, 28). It has been suggested that psychosocial factors such as trauma and intense daily psychological stress may be risk factors in individuals with genetic vulnerabilities to TS (11, 32).

The identification of parental psychopathology could be informative in the evaluation of risk factors for the development of tic disorders in children (33). Previous research has shown that there is an association between maternal psychiatric symptoms and children's tic disorders. Chronic maternal anxious symptoms and prenatal maternal depressive symptoms have been associated with increased odds of children having TS/CT at age 13 (34). In another study, a maternal history of non-specific psychiatric disorders, including anxiety disorders and depressive disorders, was shown to increase odds of children having TS/CT during childhood and adolescence (35). It is presumed that maternal depressive/anxious symptoms are associated with the occurrence of children's TS/CT *via* maternal-specific environmental and/or genetic factors (34, 35).

Although the association between maternal mental health and children's tic disorders has been proved (34, 35), it is not clear whether maternal psychiatric symptoms are associated with the subsequent course of children's tic disorders. If maternal psychiatric symptoms predict the subsequent course of children's tic disorders, then maternal psychiatric symptoms could possibly be a prognostic factor or an intervention target of tic disorders. To examine this point, we investigated the relationship between maternal depressive/anxious symptoms and children's tic frequency in early adolescence with a longitudinal design.

In addition, we speculated that maternal depressive/anxious symptoms and children's tic frequency influence each other bidirectionally. Some studies have shown bidirectional influences on maternal and children's psychiatric symptoms. For example, depression in mothers increases the risk of emotional and behavioral problems in their children and vice versa (36, 37). However, no study has investigated the bidirectional relationship between maternal psychiatric symptoms and children's tics. Research on the longitudinal bidirectional relationship between maternal depressive/anxious symptoms and children's tics would be helpful in advancing the research and practices related to tics.

Our aim was therefore to examine the relationships between maternal depressive/anxious symptoms and child's tic frequency in a longitudinal study design using a general population of early adolescent samples. In this population-based study, we referred to a tic or tics instead of the diagnostic term “tic disorders” because we did not make clinical diagnoses of the participants. Our hypotheses were as follows: (1) maternal depressive/anxious symptoms predict children's tic frequency 2 years later, and (2) maternal depressive/anxious symptoms and children's tic frequency influence each other bidirectionally over time.

## Materials and Methods

### Participants

**Figure 1:**
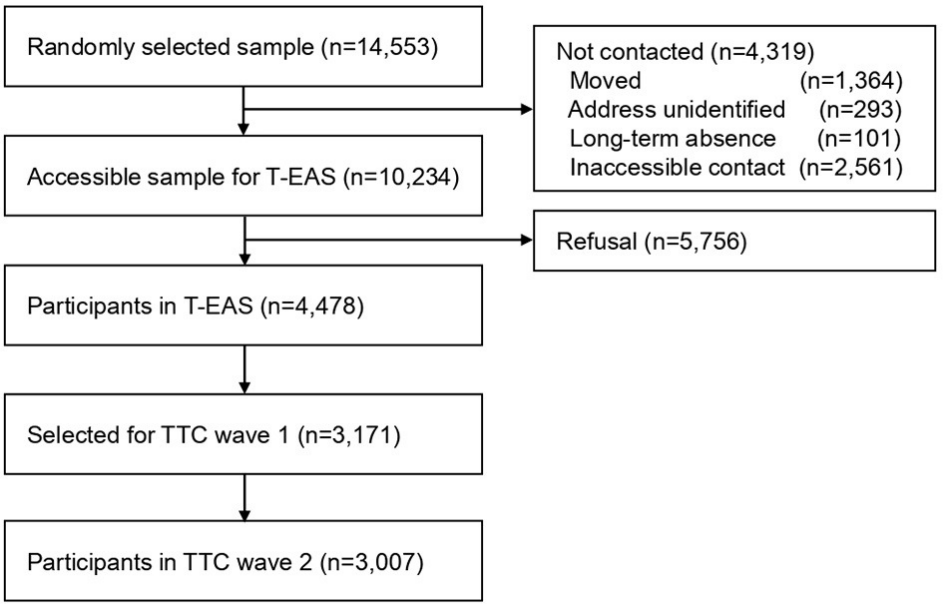
Flowchart of participant recruitment.

This study used data from the Tokyo Teen Cohort (TTC) study (http://ttcp.umin.jp/), a population-based longitudinal survey focusing on children's health from biopsychosocial multidisciplinary viewpoints (38). The TTC study has started from October 2012 and is currently being conducted. The participants were recruited from three municipalities in Tokyo (Setagaya, Mitaka, and Chofu) using the Basic Resident Register. The candidate participants were 14,553 children born between September 1, 2002, and August 31, 2004 (Figure 1). Invitation letters were sent to the primary parents of those children around their 10th birthday. Of these children, 10,234 were successfully contacted, and these children were invited to participate in the cohort study. Of these 10,234 children, 4,478 children participated in the baseline survey named the Tokyo Early Adolescence Survey (T-EAS). This baseline survey was conducted from October 2012 to January 2015, when the participants were approximately 10 years old (time 1, T1). Among the 4,478 participants in the T-EAS, candidates were chosen as participants for the second wave of the TTC study. For the sake of cohort management, the target number of participants to be included in the second wave of the TTC was 3,000 children. When choosing these TTC participants, an oversampling method was used instead of inclusion criteria, considering the low follow-up rate of families with low annual household incomes. Among children who participated in T-EAS and were interested in participating in the cohort study, all 620 children whose household annual income was lower than 4,990 thousand yen were invited. From the remaining 3,858 children, 2,551 children were randomly invited to the second wave of TTC. Thus, 3,171 participants were extracted as targets for the second wave of the TTC study. The second wave of the TTC study was carried out from August 2014 to December 2016, at the time when the participants were approximately 12 years old (time 2, T2). Of the 3,171 children who were invited, 3,007 individuals participated in the second wave of the TTC study (follow-up rate 94.8%). In each wave of the data collection, trained interviewers visited the participants' homes. They distributed questionnaires to the children and primary parents (mostly mothers), and they conducted psychological tests on the children.

### Ethical Approval

Ethical approval for this study was obtained from the research ethics committees of the Tokyo Metropolitan Institute of Medical Science (Approval Number: 12-35), The Graduate University for Advanced Studies, SOKENDAI (2012002), and the Graduate School of Medicine and Faculty of Medicine, The University of Tokyo (10057). We obtained informed assent from the children and written informed consent from their primary parents.

### Measures

#### Tic Frequency

We evaluated tic frequency at T1 and T2. The participants' primary parents answered a questionnaire about the children's tics; this questionnaire has been used in a previous study (5). The questionnaire includes a section with the following five questions about specific motor and vocal tics in the past year: “Q1: Has your child had any repeated movements of parts of the face and head (e.g., eye blinking, grimacing, sticking tongue out, licking lips, spitting)?”; “Q2: Has your child had repeated movements of the neck, shoulder or trunk (e.g., twisting around, shoulder shrugging, bending over, nodding)?”; “Q3: Has your child had repeated movements of the arms, hands, legs, or feet?”; “Q4: Has your child had repeated noises and sounds (e.g., coughing, clearing throat, grunting, gurgling, and hissing)?”; and “Q5: Has your child had repeated words or phrases?” Each question is answered as either “definitely,” “probably,” or “not at all” present. Furthermore, we asked the following question about the frequency of these repetitive behaviors: “Q6: About how often does/did this happen in the last year?” This question was answered on the following 5-point Likert scale: “1: less than once a month, 2: 1–3 times a month, 3: about once a week, 4: more than once a week, 5: every day.” We defined the participants who responded “definitely” or “probably” to any of Q1, Q2, and Q4 as having tics. The participants who only endorsed repeated movements of the arms, hands, legs or feet (Q3) or repeated words or phrases (Q5) in the absence of a positive response to the other questions about the types of tics (Q1, Q2, Q4) were excluded from a case definition to remove non-tic movements such as stereotypy or isolated echolalia. We defined as tics all responses of “definitely” or “probably” to questions concerning motor and/or vocal tics regardless of their frequency because there is no condition of frequency in the diagnostic criteria of tic disorders (1) and because we aimed to exhaustively find tics in the general population. For those without tics, the frequency of tics was regarded as 0, and for those with tics, the frequency of tics was evaluated on a 5-point scale from the answer in Q6.

#### Maternal Depressive/Anxious Symptoms

We employed the Kessler Psychological Distress Scale (K6) (39–41) for T1 and the General Health Questionnaire-28 (GHQ-28) (42, 43) for T2. The K6 and the GHQ-28 are both widely used self-report questionnaires that were developed to evaluate depressive/anxious symptoms. We used different scales between T1 and T2 in the current study because the TTC study also switched the scale used for maternal depressive/anxious symptoms from the K6 to the GHQ-28 starting at T2. The K6 is a short questionnaire consisting of 6 questions about the subjective mental distress of the respondent over the past 30 days that are answered on a 5-point scale, and the scores of the 6 items are added together (0–24 points). The GHQ-28 consists of 28 questions about the respondent's subjective physical and mental states over the past few weeks, with a total score being calculated for each item by giving 0 points each for the right two responses and 1 point each for the left two responses (0–28 points). Cutoff values are often used to screen for anxiety disorders and depression when assessing the K6 and the GHQ-28. However, in this study, we used raw values of the K6 and the GHQ-28 as continuous scales instead of screening scales, for the purpose of evaluating the severity of depressive/anxious symptoms, including the normal range in the general population. The Cronbach's alpha value was 0.84 for the K6 and 0.88 for the GHQ-28. We found that the distributions of the K6 and the GHQ-28 were similar based on the graphing cumulative distribution of their *Z* scores (Supplementary Figure 1). If a primary parent other than a mother answered the K6 or the GHQ-28, we regarded those responses as missing values.

#### Other Variables

Sex (5, 7, 44), age (32, 45, 46), maternal age (35, 47–49), socioeconomic status (50), and maternal alcohol use during pregnancy (51) were included in the analyses since previous studies have reported that these factors influence the occurrence of TS/CT. The data for these variables were obtained from the responses to the questionnaires completed by caregivers. To assess socioeconomic status, family income was evaluated on an 11-point scale, which ranged from “0–990,000 yen” to “more than 10,000,000 yen.” Information on maternal alcohol use during pregnancy was obtained from maternity record books that were provided for almost all mothers by local public organizations in Japan.

### Statistical Analysis

Longitudinal relationships between maternal depressive/anxious symptoms and children's tic frequency were studied with structural equation modeling. We used SPSS® (Statistical Package for Social Science; IBM Corp., Armonk, N.Y. USA) version 21.0 for the characteristics of the study participants and Amos ver. 22.0 (IBM Corp, New York) for the structural equation modeling. We used the following two cross-lagged design models. The first model analyzed the longitudinal relationships between maternal depressive/anxious symptoms and children's tic frequency without adjusting for covariates (unadjusted model). The second model adjusted for sex, age in months, family income, maternal age, and maternal alcohol use during pregnancy (adjusted model).

Missing values in the categories of tics, maternal depressive/anxious symptoms, and the covariates were accounted for by full information maximum likelihood procedures available in Amos. This method estimates model parameters and standard errors using all available data while adjusting for the uncertainty associated with missing data (52).

The threshold for statistical significance was set to *p* < 0.05 (two-sided) for all analyses. We evaluated the fit of our models by using the comparative fit index (CFI) and the root mean square error of approximation (RMSEA). A good model fit was indicated by an RMSEA value smaller than 0.05 and a CFI value larger than 0.95 (53, 54).

## Results

### Characteristics of the Study Participants

Table 1 shows the demographic characteristics of the 3,171 study participants. Of the 3,171 included children, 2,601 children (82.0%) had complete data about tics across both time points; 67 (2.1%) and 484 (15.3%) children had missing data about tics in either T1 or T2, respectively, and 19 (0.6%) children had missing data about tics in both T1 and T2. Across both time points, data about maternal depressive/anxious symptoms were complete for 2,683 mothers (84.6%); 24 (0.8%) and 309 (9.7%) mothers had missing scores in either T1 or T2, respectively; and 155 mothers (4.9%) had missing data about maternal depressive/anxious symptoms in both T1 and T2.

**Table 1:** Demographic characteristics of the participants.

**Variables**	**T1** **(10 years of age)**		**T2** **(12 years of age)**	
	**n/*Mean***	**(%)/*SD***	**n/*Mean***	**(%)/*SD***
Sex, male	1,684	(53.1%)		
Age in months	*122.1*	*3.3*	*146.0*	*3.7*
Maternal age	*42.0*	*4.2*		
Family income^a^				
<5 million yen	620	(20.4%)	448	(16.5%)
≥5 million yen, <8 million yen	941	(31.0%)	782	(28.8%)
≥8 million yen, <10 million yen	568	(18.6%)	518	(19.1%)
≥10 million yen	917	(30.1%)	970	(35.7%)
Maternal alcohol use during pregnancy	748	(27.2%)		
**Tic frequency**				
No tics	2,365	(76.7%)	2,042	(76.5%)
With tics	720	(23.3%)	626	(23.5%)
Less than once a month	138	(4.5%)	122	(4.6%)
1–3 times a month	114	(3.7%)	115	(4.3%)
About once a week	37	(1.2%)	66	(2.5%)
More than once a week	206	(6.7%)	161	(6.0%)
Every day	225	(7.3%)	162	(6.1%)
**Maternal depressive/anxious symptoms**				
T1, K6	*2.9*	*3.3*		
T2, GHQ-28			*5.4*	*4.9*

a *Family income was evaluated on the 11-point scale described in section Materials and Method and categorized into the four groups in this table*.

*SD, standard deviation; K6, Kessler Psychological Distress Scale; GHQ-28, General Health Questionnaire-28*.

Of the participants, 23.3% children (720 of 3,085 available data) at T1 and 23.5% children (626 of 2,668 available data) at T2 had tics. These prevalence rates are consistent with previous studies that have estimated the point prevalence of tics in childhood to be approximately 20–29% (2–4). Of the 2,601 people whose data on the presence of tics were obtained in both T1 and T2, 332 participants endorsed tics at both T1 and T2, 280 participants endorsed tics only for T1, 265 participants endorsed tics for only T2, and 1,724 did not endorse tics at either time point.

### Longitudinal Relationships Between Maternal Depressive/Anxious Symptoms and Children's Tic Frequency in Early Adolescence

**Figure 2:**
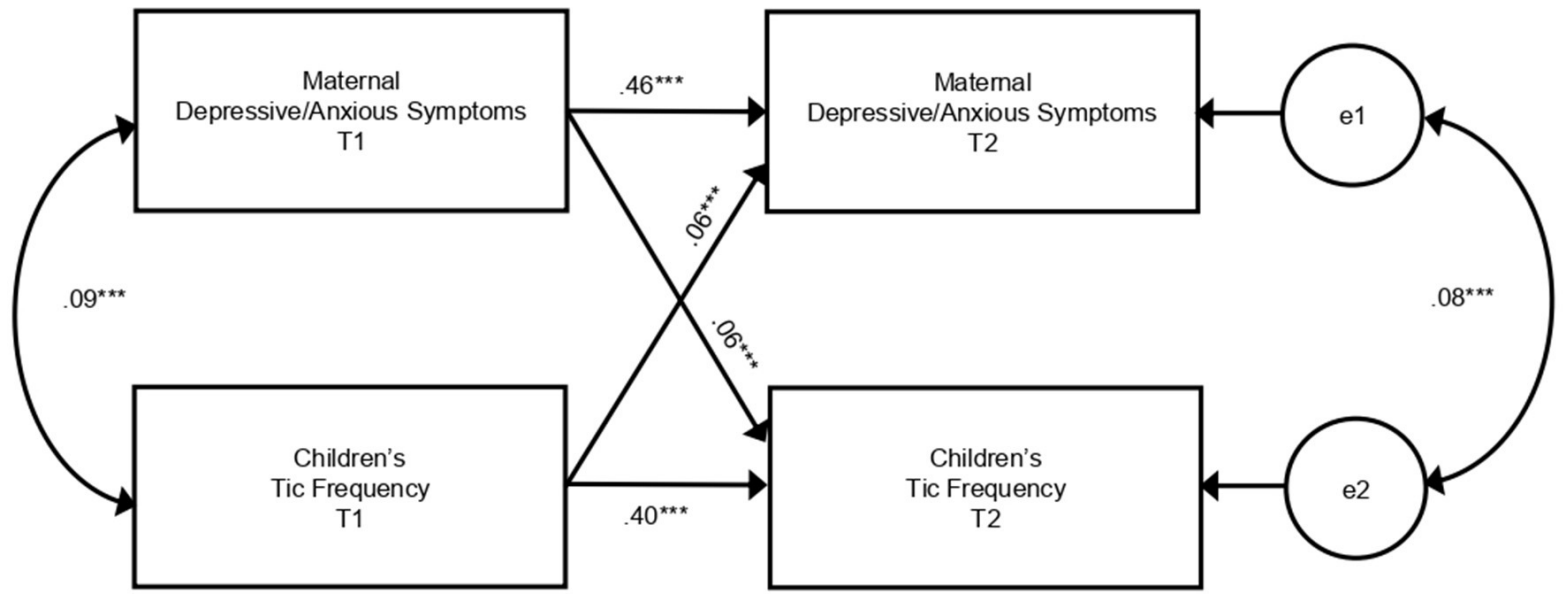
Cross-lagged model of relationships between maternal depressive/anxious symptoms and children's tic frequency. This figure shows the results of the adjusted model in Table 2. Paths from covariates are omitted from the figure. e, error variable; T1, 10 years of age; T2, 12 years of age. ***p < 0.001.

We investigated the relationships between maternal depressive/anxious symptoms and children's tic frequency in a cross-lagged model analysis (Figure 2, Table 2). There was a cross-sectional association between maternal depressive/anxious symptoms and child's tic frequency at T1 and T2. Higher levels of maternal depressive/anxious symptoms at T1 significantly increased children's tic frequency at T2 (adjusted model: β = 0.06, *p* < 0.001). In contrast, higher frequency of children's tics at T1 was related to higher levels of maternal depressive/anxious symptoms at T2 (adjusted model: β = 0.06, *p* < 0.001). All of these models indicated good model fit to the data (adjusted model: CFI = 0.950, RMSEA = 0.046). These results revealed that maternal depressive/anxious symptoms and children's tic frequency had longitudinal, bidirectional relationships with each other.

**Table 2:** Relationships between maternal depressive/anxious symptoms and children's tic frequency (n = 3,171).

	**Unadjusted model**					**Adjusted model**				
**Path**	**β**	**B**	**SE**	**95% CI**	***p-*value**	**β**	**B**	**SE**	**95% CI**	***p-*value**
Maternal depressive/anxious symptoms T1 ⇔ Children's tic frequency T1	0.10	0.50	0.10	0.31–0.70	<0.001	0.09	0.49	0.10	0.30–0.69	<0.001
Maternal depressive/anxious symptoms T2 ⇔ Children's tic frequency T2	0.09	0.52	0.12	0.28–0.75	<0.001	0.08	0.50	0.12	0.26–0.73	<0.001
Maternal depressive/anxious symptoms T1 → Maternal depressive/anxious symptoms T2	0.47	0.69	0.03	0.09–0.74	<0.001	0.46	0.68	0.03	0.63–0.73	<0.001
Children's tic frequency t1 → Children's tic frequency T2	0.41	0.39	0.02	0.35–0.42	<0.001	0.40	0.38	0.02	0.35–0.41	<0.001
Maternal depressive/anxious symptoms T1 → Children's tic frequency T2	0.06	0.03	0.01	0.01–0.05	<0.001	0.06	0.03	0.01	0.01–0.04	<0.001
Children's tic frequency T1 → Maternal depressive/anxious symptoms T2	0.07	0.20	0.05	0.10–0.30	<0.001	0.06	0.19	0.05	0.09–0.30	<0.001
	χ^2^ = 0, Degrees of freedom = 0, *p* = -, CFI = 1.0, RMSEA = 0 (saturated model)					χ^2^ = 77.834, Degrees of freedom = 10, *p* < 0.001, CFI = 0.950, RMSEA = 0.046				

*T1, 10 years of age; T2, 12 years of age; β, standardized coefficient; B, coefficient; SE, standard error; CI, confidence interval; CFI, comparative fit index; RMSEA, root mean square error of approximation*.

## Discussion

This was the first study to examine the longitudinal relationships between maternal depressive/anxious symptoms and children's tic frequency in a population-based early adolescent sample. The following two findings were obtained. First, when more severe maternal depressive/anxious symptoms are present, children are likely to present more frequent tics 2 years later. Second, the severity of maternal depressive/anxious symptoms and children's tic frequency are longitudinally associated with each other.

The present results showed cross-sectional relationship between maternal anxiety/depressive symptoms and children's tic frequency and small but significant longitudinal bidirectional relationship between them. Previous studies showed relationship between past maternal mental symptoms and children's TS/CT, and presumed that maternal mental symptoms are associated with the occurrence of children's TS/CT *via* maternal-specific environmental and/or genetic factors (34, 35). The present results is consistent with those previous studies and provided a new perspective that suggests longitudinal bidirectional relationship between maternal anxiety/depressive symptoms and children's tic frequency.

Several explanations may be possible for the significant magnitude-response relationship between maternal depressive/anxious symptoms and children's later tic frequency. First, maternal depressive/anxious symptoms may affect the occurrence, persistence, and exacerbation of children's tics as an environmental factor because environmental factors such as psychosocial stresses are known to exacerbate tics (55–59). In addition, several studies have shown that tic frequency can be influenced by antecedent environmental events and social consequences although they referred relatively immediate and short term reaction. For example, some activities reduce tic frequency such as focusing attention away from tics (60), aerobic exercise training (61) and participation in musical activity (62). There are no reports of an association between maternal psychiatric symptoms and the course of children's tics. However, some studies have shown an association between maternal psychiatric symptoms and the course of children's psychiatric symptoms. For example, maternal depression has been shown to be associated with increased psychiatric diagnoses and emotional and behavioral problems in children, and when maternal depression is remitted, the children's problems are more likely to also be in remission (63). Furthermore, improvement in parental depression has a positive impact on the health, emotional, cognitive, academic and overall functioning of children (64). Thus, similar to these reports, maternal depressive/anxious symptoms might affect the course of children's tics as an environmental factor. Second, there might be genetic relationships between maternal depressive/anxious symptoms and the occurrence, persistence, and exacerbation of tics. Family studies of TS have suggested that TS has genetic correlations with depressive disorders and anxiety disorders (65, 66), although these correlations are possibly mediated through ADHD and OCD (66). There has been no research examining the genetic relationships between maternal depressive/anxious symptoms and the persistence or exacerbation of tics, but the results of this research could not rule out these possibilities. Furthermore, there is also a possibility that an interaction of genetic and environmental factors is involved in the relationships between maternal depressive/anxious symptoms and children's tics. The results of this study could not distinguish genetic and environmental contributors to the relationships between maternal depressive/anxious symptoms and children's tics.

Our findings on the association between children's tic frequency and increased maternal depressive/anxious symptoms 2 years later can be explained by several potential mechanisms. One of the possible mechanisms is that the parenting stress associated with bringing up children with tics might influence maternal depressive/anxious symptoms. Parents of children with TS experience increased levels of caregiver burdens and parenting stress compared to parents of children without TS (67, 68). The visible nature of tics can have an impact on the parent-child relationship, with parents becoming overprotective of, worrying about, struggling to accept or trying to control children's tics, which can lead to family conflicts, poor parent-child relationships and increased frustrations in parenting (7, 9, 10, 69, 70). Parenting stress in parents of children with tics could also occur due to children's comorbidities, such as ADHD, OCD, and behavioral problems (67, 68). In a previous population-based study, 21.2% of children had tics, and children with tics were more affected by psychopathologies, including ADHD and OCD, than were children without tics (12). In addition to these environmental factors, both genetic factors and genetic/environmental interactions might have an effect of children's tics on maternal depressive/anxious symptoms.

The implications of this study were that the longitudinal bidirectional relationships between maternal depressive/anxious symptoms and children's tic frequency may suggest a vicious cycle in which maternal depressive/anxious symptoms make tic frequency increased, and children's tic frequency make maternal depressive/anxious symptoms worse. This study also suggested that not only intervention in children's tics but also intervention in maternal depressive/anxious symptoms might be important for the treatment of tics. However, the present study was unable to separate genetic and environmental factors in the association between children's tic frequency and maternal depressive/anxious symptoms; therefore, further research is needed to determine the effect of intervention on maternal anxiety/depressive symptoms. While there has been a consensus on the importance of family psychoeducation in the treatment of tics (7, 27), it is not known whether maternal psychiatric problems influence the course of children's tics. This study provides new insights for future research and practice.

The strength of this study was that, for the first time, it was shown that higher levels of maternal depressive/anxious symptoms are related to an increased children's tic frequency 2 years later and that there are longitudinal relationships between maternal depressive/anxious symptoms and children's tic frequency. Other strengths were the large sample size, the high follow-up rate of the study, and the inclusion of non-clinical tics. In contrast, this study also had several limitations. First, we used different measures of maternal depressive/anxious symptoms for T1 and T2. We found that the distributions of the K6 and the GHQ-28 were similar by graphing the cumulative distribution of the *Z* scores of the K6 and the GHQ-28 (Supplementary Figure 1). Additionally, this limitation did not influence the course from maternal depressive/anxious symptoms to children's tic frequency. Second, in this study, children's tics were evaluated not by direct clinical assessments but by questionnaires to caregivers. However, we deliberately chose rigorous tic definitions and sought to exclude participants with non-tic movement disorders (e.g., stereotypies associated with autism or an intellectual disability, repetitive arm/leg movements that could be better explained by tremor, or motor restlessness) (5). In this study, the prevalence of tics was 23.3% at age 10 and 23.5% at age 12. These prevalence rates could be considered reasonable based on the following evidence. Point prevalence depends strongly on age; the highest rate is estimated to be approximately 20% at age 5–10, and the lifetime prevalence is much higher (3). In previous studies that have directly observed children, tics were found in 29.2% of fourth-grade children in an elementary school in Washington D.C. (2) and in 21.2% of children aged 9–17 years old (mean 13.1 years old) in Monroe County, Rochester, New York (4). The prevalence rates of tics in the present study were consistent with those found in these previous studies. Third, the data analysis in this study could not adjust for ADHD and OCD, which are frequently comorbid with tics. That may be because of the strong association of tics with ADHD and OCD. Future studies are needed to examine the effects of ADHD and OCD on the bidirectional relationships between maternal depressive/anxious symptoms and children's tic frequency. The fourth limitation was that the research interval in this longitudinal study was relatively short. Typically, tics improve gradually during adolescence, with repeated periods of remission and exacerbation. Thus, it might be difficult to capture change in the short research period of 2 years. Longer-term follow-up periods are needed in the future. The fifth limitation was that we did not collect information about the maternal history of tics. Given the low rate of medical consultation for tics (5, 12, 65) and the clinical outcome that tics often improve or disappear after adolescence (7, 13, 14), it is probably not possible to obtain accurate information on the maternal history of tics. Finally, there were also some limitations inherent to the cross-lagged model (71); i.e., there is a possibility that there are multiple potential additional factors (not included in the model) that influence the bidirectional relationship over time.

The following two studies would be helpful in testing the viability of the relationships between maternal depressive/anxious symptoms and children's tic frequency and in advancing research and practice. First, if the course of children's tic frequency could be observed at three or more time points, it would be possible to confirm the vicious cycle that develops between maternal depressive/anxious symptoms and children's tics and to investigate the mediating factors. For example, children's anxiety/depression symptoms might mediate the relationships between maternal depressive/anxious symptoms and children's tic frequency. In addition, comorbidities or poor quality of life might mediate the relationships between children's tic frequency and maternal depressive/anxious symptoms. Second, intervention studies could examine whether improvements in maternal depressive/anxious symptoms improve children's tics.

This study was the first to show the relationship between preceding maternal depressive/anxious symptoms and an increased children's tic frequency 2 years later in early adolescence. Furthermore, we found longitudinal bidirectional relationships between maternal depressive/anxious symptoms and children's tic frequency. Although we could not separate environmental factors and genetic factors in this research, the findings implied that it may be important to care not only for children with tics but also for their mothers' depressive/anxious symptoms when tics are treated in early adolescence.

## Data Availability Statement

The raw data supporting the conclusions of this article will be made available by the authors, without undue reservation.

## Ethics Statement

The studies involving human participants were reviewed and approved by the Tokyo Metropolitan Institute of Medical Science (Approval Number: 12-35), the Graduate University for Advanced Studies, SOKENDAI (2012002), the Graduate School of Medicine and Faculty of Medicine, the University of Tokyo (10057). Written informed consent to participate in this study was provided by the participants' legal guardian/next of kin.

## Author Contributions

TY conceptualized and designed the study, drafted the initial manuscript, reviewed and revised the manuscript, and being supervised by SA. AN, SY, YK, and KK critically reviewed the manuscript for important intellectual content and contributed to the discussion. SU supervised the statistical analysis. All authors contributed to and have approved the final manuscript.

## Funding

This work was supported by Grant-in-Aid for Scientific Research on Innovative Areas (JP23118002 and JP16H01689; Adolescent Mind and Self-Regulation) from the Ministry of Education, Culture, Sports, Science, and Technology of Japan. This study was also supported in part by JSPS KAKENHI Grant Numbers JP16H06395, JP16H06398, JP16H06399, JP16K15566, JP16K21720, JP17H05931, and JP19K17055, by UTokyo Center for Integrative Science of Human Behavior (CiSHuB), by the International Research Center for Neurointelligence (WPI-IRCN) at the University of Tokyo Institutes for Advanced Study (UTIAS), and by AMED under Grant Number JP17ek0109262. The funding source had no role in the preparation, review, or approval of the manuscript, and the decision to submit the manuscript for publication.

## Conflict of Interest

The authors declare that the research was conducted in the absence of any commercial or financial relationships that could be construed as a potential conflict of interest.

## Publisher's Note

All claims expressed in this article are solely those of the authors and do not necessarily represent those of their affiliated organizations, or those of the publisher, the editors and the reviewers. Any product that may be evaluated in this article, or claim that may be made by its manufacturer, is not guaranteed or endorsed by the publisher.

## Abbreviations

ADHD: attention-deficit/hyperactivity disorder
CFI: comparative fit index
CI: confidence interval
CT: chronic tic disorder
DSM-5: Diagnostic and Statistical Manual of Mental Disorders 5th edition
GHQ-28: General Health Questionnaire-28
K6: Kessler Psychological Distress Scale
OCD: obsessive-compulsive disorder
RMSEA: root mean square error of approximation
SD: standard deviation
TS: Tourette syndrome
TTC: Tokyo Teen Cohort.

## References

[1] American Psychiatric Association. The Diagnostic and Statistical Manual of Mental Disorders. 5th ed. Washington, DC: American Psychiatric Publishing (2013).

[2] Snider LA Seligman LD Ketchen BR Levitt SJ Bates LR Garvey MA. Tics and problem behaviors in schoolchildren: prevalence, characterization, and associations. Pediatrics. (2002) 110:331–6. 10.1542/peds.110.2.331 12165586

[3] Black KJ Black ER Greene DJ Schlaggar BL. Provisional tic disorder: what to tell parents when their child first starts ticcing. F1000Res. (2016) 5:696. 10.12688/f1000research.8428.1 27158458 PMC4850871

[4] Kurlan R McDermott MP Deeley C Como PG Brower C Eapen S. Prevalence of tics in schoolchildren and association with placement in special education. Neurology. (2001) 57:1383–8. 10.1212/WNL.57.8.1383 11673576

[5] Scharf JM Miller LL Mathews CA Ben-Shlomo Y. Prevalence of Tourette syndrome and chronic tics in the population-based Avon longitudinal study of parents and children cohort. J Am Acad Child Adolesc Psychiatry. (2012) 51:192–201.e5. 10.1016/j.jaac.2011.11.004 22265365 PMC3314954

[6] World Health Organization. Tenth Revision of the International Classification of Diseases and Related Health Problems. Geneva: World Health Organization (1992).

[7] Murphy TK Lewin AB Storch EA Stock S. Practice parameter for the assessment and treatment of children and adolescents with tic disorders. J Am Acad Child Adolesc Psychiatry. (2013) 52:1341–59. 10.1016/j.jaac.2013.09.015 24290467

[8] Hoekstra PJ Lundervold AJ Lie SA Gillberg C Plessen KJ. Emotional development in children with tics: a longitudinal population-based study. Eur Child Adolesc Psychiatry. (2013) 22:185–92. 10.1007/s00787-012-0337-y 23064999

[9] Smith H Fox JR Trayner P. The lived experiences of individuals with Tourette syndrome or tic disorders: a meta-synthesis of qualitative studies. Br J Psychol. (2015) 106:609–34. 10.1111/bjop.12118 25721405

[10] Malli MA Forrester-Jones R Murphy G. Stigma in youth with Tourette's syndrome: a systematic review and synthesis. Eur Child Adolesc Psychiatry. (2016) 25:127–39. 10.1007/s00787-015-0761-x 26316059

[11] Leckman JF. Tourette's syndrome. Lancet. (2002) 360:1577–86. 10.1016/S0140-6736(02)11526-1 12443611

[12] Kurlan R Como PG Miller B Palumbo D Deeley C Andresen EM. The behavioral spectrum of tic disorders: a community-based study. Neurology. (2002) 59:414–20. 10.1212/WNL.59.3.414 12177376

[13] Leckman JF Bloch MH King RA Scahill L. Phenomenology of tics and natural history of tic disorders. Adv Neurol. (2006) 99:1–16. 16536348

[14] Bloch MH Leckman JF. Clinical course of Tourette syndrome. J Psychosom Res. (2009) 67:497–501. 10.1016/j.jpsychores.2009.09.002 19913654 PMC3974606

[15] Kurlan R Behr J Medved L Como P. Transient tic disorder and the spectrum of Tourette's syndrome. Arch Neurol. (1988) 45:1200–1. 10.1001/archneur.1988.00520350038012 3190500

[16] Kurlan R Eapen V Stern J McDermott MP Robertson MM. Bilineal transmission in Tourette's syndrome families. Neurology. (1994) 44:2336–42. 10.1212/WNL.44.12.2336 7991122

[17] Peterson BS Pine DS Cohen P Brook JS. Prospective, longitudinal study of tic, obsessive-compulsive, and attention-deficit/hyperactivity disorders in an epidemiological sample. J Am Acad Child Adolesc Psychiatry. (2001) 40:685–95. 10.1097/00004583-200106000-00014 11392347

[18] Muller-Vahl KR Sambrani T Jakubovski E. Tic disorders revisited: introduction of the term “tic spectrum disorders”. Eur Child Adolesc Psychiatry. (2019) 28:1129–35. 10.1007/s00787-018-01272-7 30661132 PMC6675752

[19] Conelea CA Woods DW. The influence of contextual factors on tic expression in Tourette's syndrome: a review. J Psychosom Res. (2008) 65:487–96. 10.1016/j.jpsychores.2008.04.010 18940379

[20] Peterson AL Azrin NH. An evaluation of behavioral treatments for Tourette syndrome. Behav Res Ther. (1992) 30:167–74. 10.1016/0005-7967(92)90140-C 1567346

[21] Woods DW Twohig MP Flessner CA Roloff TJ. Treatment of vocal tics in children with Tourette syndrome: investigating the efficacy of habit reversal. J Appl Behav Anal. (2003) 36:109–12. 10.1901/jaba.2003.36-109 12723873 PMC1284423

[22] Woods DW Himle MB. Creating tic suppression: comparing the effects of verbal instruction to differential reinforcement. J Appl Behav Anal. (2004) 37:417–20. 10.1901/jaba.2004.37-417 15529900 PMC1284518

[23] Azrin NH Peterson AL. Habit reversal for the treatment of Tourette syndrome. Behav Res Ther. (1988) 26:347–51. 10.1016/0005-7967(88)90089-7 3214400

[24] Espil FM Capriotti MR Conelea CA Woods DW. The role of parental perceptions of tic frequency and intensity in predicting tic-related functional impairment in youth with chronic tic disorders. Child Psychiatry Hum Dev. (2014) 45:657–65. 10.1007/s10578-013-0434-2 24395287 PMC4085134

[25] Cavanna AE Servo S Monaco F Robertson MM. The behavioral spectrum of Gilles de la Tourette syndrome. J Neuropsychiatry Clin Neurosci. (2009) 21:13–23. 10.1176/jnp.2009.21.1.13 19359447

[26] Mataix-Cols D Isomura K Perez-Vigil A Chang Z Ruck C Larsson KJ. Familial risks of Tourette syndrome and chronic tic disorders. A population-based cohort study. JAMA Psychiatry. (2015) 72:787–93. 10.1001/jamapsychiatry.2015.0627 26083307

[27] Kurlan R. Clinical practice. Tourette's syndrome. N Engl J Med. (2010) 363:2332–8. 10.1056/NEJMcp1007805 21142535

[28] Cavanna AE Seri S. Tourette's syndrome. BMJ. (2013) 347:f4964. 10.1136/bmj.f4964 23963548

[29] Felling RJ Singer HS. Neurobiology of tourette syndrome: current status and need for further investigation. J Neurosci. (2011) 31:12387–95. 10.1523/JNEUROSCI.0150-11.2011 21880899 PMC6703258

[30] Kalanithi PS Zheng W Kataoka Y DiFiglia M Grantz H Saper CB. Altered parvalbumin-positive neuron distribution in basal ganglia of individuals with Tourette syndrome. Proc Natl Acad Sci USA. (2005) 102:13307–12. 10.1073/pnas.0502624102 16131542 PMC1201574

[31] Wang Z Maia TV Marsh R Colibazzi T Gerber A Peterson BS. The neural circuits that generate tics in Tourette's syndrome. Am J Psychiatry. (2011) 168:1326–37. 10.1176/appi.ajp.2011.09111692 21955933 PMC4246702

[32] Rutter M Bishop D Pine D Scott S Stevenson J Taylor E. eds. Rutter's Child and Adolescent Psychiatry. 5th ed. London: Blackwell Press (2010).

[33] Coffey BJ. Persistent tics, Tourette syndrome, and psychopathology: where are we now, and where are we going? J Am Acad Child Adolesc Psychiatry. (2017) 56:281–3. 10.1016/j.jaac.2017.01.015 28335870

[34] Ben-Shlomo Y Scharf JM Miller LL Mathews CA. Parental mood during pregnancy and post-natally is associated with offspring risk of Tourette syndrome or chronic tics: prospective data from the Avon Longitudinal Study of Parents and Children (ALSPAC). Eur Child Adolesc Psychiatry. (2016) 25:373–81. 10.1007/s00787-015-0742-0 26174227 PMC4820468

[35] Leivonen S Scharf JM Mathews CA Chudal R Gyllenberg D Sucksdorff D. Parental psychopathology and Tourette syndrome/chronic tic disorder in offspring: a nationwide case-control study. J Am Acad Child Adolesc Psychiatry. (2017) 56:297–303.e4. 10.1016/j.jaac.2017.01.009 28335873

[36] Elgar FJ McGrath PJ Waschbusch DA Stewart SH Curtis LJ. Mutual influences on maternal depression and child adjustment problems. Clin Psychol Rev. (2004) 24:441–59. 10.1016/j.cpr.2004.02.002 15245830

[37] Kuckertz JM Mitchell C Wiggins JL. Parenting mediates the impact of maternal depression on child internalizing symptoms. Depress Anxiety. (2018) 35:89–97. 10.1002/da.22688 28962070 PMC5760303

[38] Ando S Nishida A Yamasaki S Koike S Morimoto Y Hoshino A. Cohort profile: the Tokyo teen cohort study (TTC). Int J Epidemiol. (2019) 48:1414.g. 10.1093/ije/dyz033 30879075 PMC6857749

[39] Sakurai K Nishi A Kondo K Yanagida K Kawakami N. Screening performance of K6/K10 and other screening instruments for mood and anxiety disorders in Japan. Psychiatry Clin Neurosci. (2011) 65:434–41. 10.1111/j.1440-1819.2011.02236.x 21851452

[40] Furukawa TA Kawakami N Saitoh M Ono Y Nakane Y Nakamura Y. The performance of the Japanese version of the K6 and K10 in the World Mental Health Survey Japan. Int J Methods Psychiatr Res. (2008) 17:152–8. 10.1002/mpr.257 18763695 PMC6878390

[41] Kessler RC Berglund P Demler O Jin R Merikangas KR Walters EE. Lifetime prevalence and age-of-onset distributions of DSM-IV disorders in the National Comorbidity Survey Replication. Arch Gen Psychiatry. (2005) 62:593–602. 10.1001/archpsyc.62.6.593 15939837

[42] Goldberg DP. The detection of psychiatric illness by questionnaire. In: A Technique for the Identification and Assessment of Non-psychotic Psychiatric Illness. London: Oxford University Press (1972).

[43] Goldberg DP Hillier VF. A scaled version of the general health questionnaire. Psychol Med. (1979) 9:139–45. 10.1017/S0033291700021644 424481

[44] Stefanoff P Wolanczyk T Gawrys A Swirszcz K Stefanoff E Kaminska A. Prevalence of tic disorders among schoolchildren in Warsaw, Poland. Eur Child Adolesc Psychiatry. (2008) 17:171–8. 10.1007/s00787-007-0651-y 17876501

[45] Leckman JF Zhang H Vitale A Lahnin F Lynch K Bondi C. Course of tic severity in Tourette syndrome: the first two decades. Pediatrics. (1998) 102:14–9. 10.1542/peds.102.1.14 9651407

[46] Leckman JF. Phenomenology of tics and natural history of tic disorders. Brain Dev. (2003) 25(Suppl. 1):S24–8. 10.1016/S0387-7604(03)90004-0 14980368

[47] Leivonen S Voutilainen A Chudal R Suominen A Gissler M Sourander A. Obstetric and neonatal adversities, parity, and Tourette syndrome: a nationwide registry. J Pediatr. (2016) 171:213–9. 10.1016/j.jpeds.2015.10.063 26608088

[48] Leivonen S Chudal R Joelsson P Ekblad M Suominen A Brown AS. Prenatal maternal smoking and Tourette syndrome: a nationwide register study. Child Psychiatry Hum Dev. (2016) 47:75–82. 10.1007/s10578-015-0545-z 25796373

[49] McGrath JJ Petersen L Agerbo E Mors O Mortensen PB Pedersen CB. comprehensive assessment of parental age and psychiatric disorders. JAMA psychiatry. (2014) 71:301–9. 10.1001/jamapsychiatry.2013.4081 24452535

[50] Miller LL Scharf JM Mathews CA Ben-Shlomo Y. Tourette syndrome and chronic tic disorder are associated with lower socio-economic status: findings from the Avon Longitudinal Study of Parents and Children cohort. Dev Med Child Neurol. (2014) 56:157–63. 10.1111/dmcn.12318 24138188 PMC3908357

[51] Mathews CA Scharf JM Miller LL Macdonald-Wallis C Lawlor DA Ben-Shlomo Y. Association between pre- and perinatal exposures and Tourette syndrome or chronic tic disorder in the ALSPAC cohort. Br J Psychiatry. (2014) 204:40–5. 10.1192/bjp.bp.112.125468 24262815 PMC3877832

[52] Schafer JL Graham JW. Missing data: our view of the state of the art. Psychol Methods. (2002) 7:147–77. 10.1037/1082-989X.7.2.147 12090408

[53] Byrne BM. Structural Equation Modeling with AMOS: Basic Concepts, Applications, and Programming. 2nd ed. New York, NY: Routledge (2010).

[54] Kline RB. Principles and Practice of Structural Equation Modeling. 2nd ed. New York, NY: The Guilford (2005).

[55] Lin H Katsovich L Ghebremichael M Findley DB Grantz H Lombroso PJ. Psychosocial stress predicts future symptom severities in children and adolescents with Tourette syndrome and/or obsessive-compulsive disorder. J Child Psychol Psychiatry. (2007) 48:157–66. 10.1111/j.1469-7610.2006.01687.x 17300554 PMC3073143

[56] Hoekstra PJ Steenhuis MP Kallenberg CG Minderaa RB. Association of small life events with self reports of tic severity in pediatric and adult tic disorder patients: a prospective longitudinal study. J Clin Psychiatry. (2004) 65:426–31. 10.4088/JCP.v65n0320 15096084

[57] Chappell P Riddle M Anderson G Scahill L Hardin M Walker D. Enhanced stress responsivity of Tourette syndrome patients undergoing lumbar puncture. Biol Psychiatry. (1994) 36:35–43. 10.1016/0006-3223(94)90060-4 8080901

[58] Silva RR Munoz DM Barickman J Friedhoff AJ. Environmental factors and related fluctuation of symptoms in children and adolescents with Tourette's disorder. J Child Psychol Psychiatry. (1995) 36:305–12. 10.1111/j.1469-7610.1995.tb01826.x 7759592

[59] Surwillo WW Shafii M Barrett CL. Gilles de la Tourette syndrome: a 20-month study of the effects of stressful life events and haloperidol on symptom frequency. J Nerv Ment Dis. (1978) 166:812–6. 10.1097/00005053-197811000-00011 281456

[60] Misirlisoy E Brandt V Ganos C Tubing J Munchau A Haggard P. The relation between attention and tic generation in Tourette syndrome. Neuropsychology. (2015) 29:658–65. 10.1037/neu0000161 25486384 PMC4484548

[61] Jackson GM Nixon E Jackson SR. Tic frequency and behavioural measures of cognitive control are improved in individuals with Tourette syndrome by aerobic exercise training. Cortex. (2020) 129:188–98. 10.1016/j.cortex.2020.01.029 32492517

[62] Bodeck S Lappe C Evers S. Tic-reducing effects of music in patients with Tourette's syndrome: self-reported and objective analysis. J Neurol Sci. (2015) 352:41–7. 10.1016/j.jns.2015.03.016 25805454

[63] Weissman MM Pilowsky DJ Wickramaratne PJ Talati A Wisniewski SR Fava M. Remissions in maternal depression and child psychopathology: a STAR^*^D-child report. JAMA. (2006) 295:1389–98. 10.1001/jama.295.12.1389 16551710

[64] Gunlicks ML Weissman MM. Change in child psychopathology with improvement in parental depression: a systematic review. J Am Acad Child Adolesc Psychiatry. (2008) 47:379–89. 10.1097/CHI.0b013e3181640805 18388766

[65] Khalifa N von Knorring AL. Tourette syndrome and other tic disorders in a total population of children: clinical assessment and background. Acta Paediatr. (2005) 94:1608–14. 10.1111/j.1651-2227.2005.tb01837.x 16352498

[66] Hirschtritt ME Lee PC Pauls DL Dion Y Grados MA Illmann C. Lifetime prevalence, age of risk, and genetic relationships of comorbid psychiatric disorders in Tourette syndrome. JAMA psychiatry. (2015) 72:325–33. 10.1001/jamapsychiatry.2014.2650 25671412 PMC4446055

[67] Stewart SB Greene DJ Lessov-Schlaggar CN Church JA Schlaggar BL. Clinical correlates of parenting stress in children with Tourette syndrome and in typically developing children. J Pediatr. (2015) 166:1297–302.e3. 10.1016/j.jpeds.2015.01.041 25769235 PMC4414884

[68] Cooper C Robertson MM Livingston G. Psychological morbidity and caregiver burden in parents of children with Tourette's disorder and psychiatric comorbidity. J Am Acad Child Adolesc Psychiatry. (2003) 42:1370–5. 10.1097/01.CHI.0000085751.71002.48 14566175

[69] Elstner K Selai CE Trimble MR Robertson MM. Quality of life (QOL) of patients with Gilles de la Tourette's syndrome. Acta Psychiatr Scand. (2001) 103:52–9. 10.1111/j.1600-0447.2001.00147.x 11202129

[70] Eddy CM Rizzo R Gulisano M Agodi A Barchitta M Cali P. Quality of life in young people with Tourette syndrome: a controlled study. J Neurol. (2011) 258:291–301. 10.1007/s00415-010-5754-6 20859745

[71] Selig JP Little TD. Autoregressive and cross-lagged panel analysis for longitudinal data. In: Laursen B, Little TD, Card NA, editors. Handbook of Developmental Research Methods. New York, NY: The Guilford Press (2012). p. 265–78.

[72] Tomoko Y Shuntaro A Satoshi U Syudo Y Masaya M Tomoki K. Longitudinal bidirectional relationships between maternal depressive/anxious symptoms and children's tic frequency in early adolescence. Research Square [Preprint] (2021). 10.21203/rs.3.rs-17370/v3 PMC8652242 34899427

