# Supplementary figures and images for: Longitudinal Bidirectional Relationships Between Maternal Depressive/Anxious Symptoms and Children's Tic Frequency in Early Adolescence
Source: Front Psychiatry. 2021 Nov 24;12:767571. doi: 10.3389/fpsyt.2021.767571 (PMC8652242; doi:10.3389/fpsyt.2021.767571)

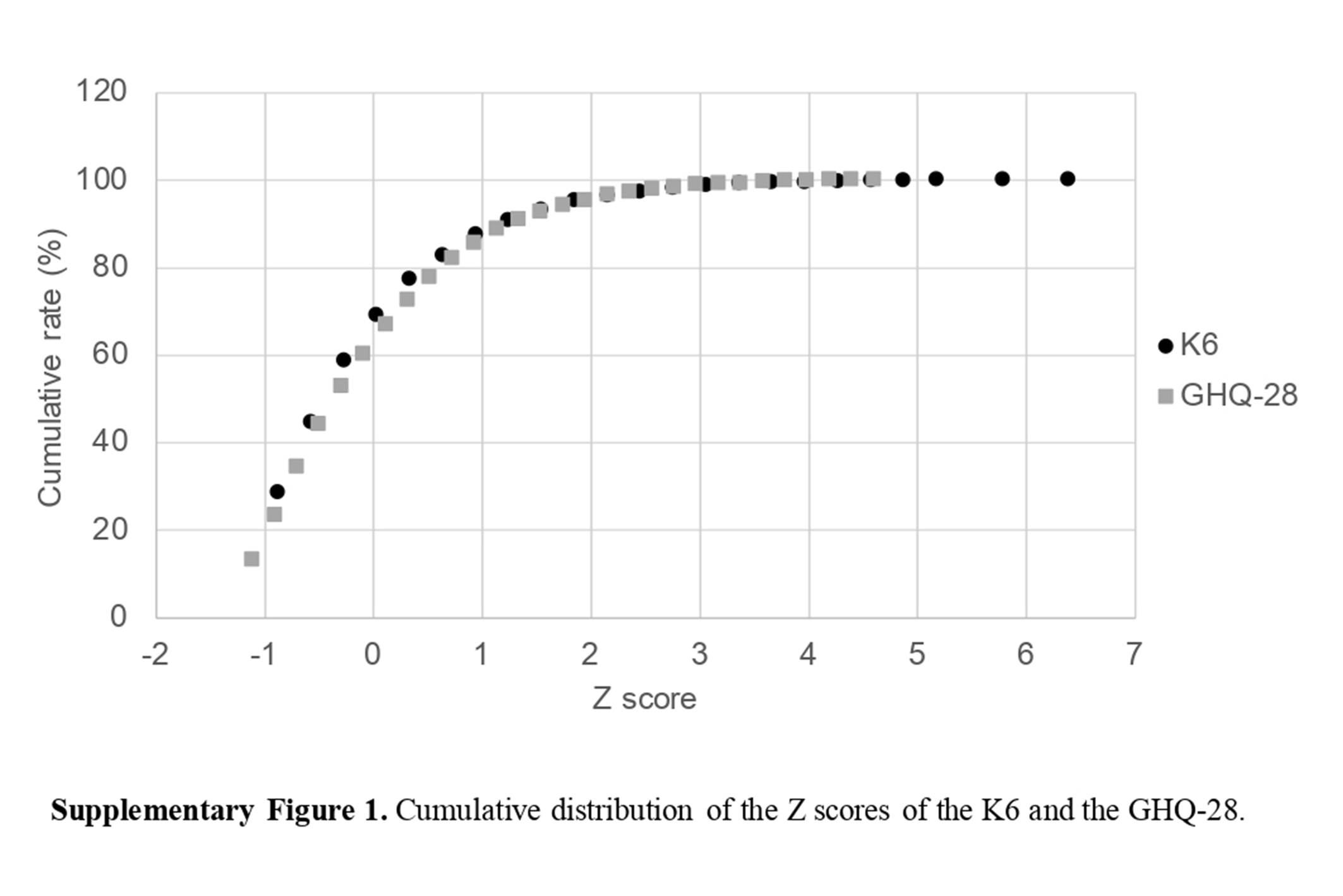

Supplement: Supplementary file 1 [file Image_1.jpg]
